# Serum vitamin D levels and type 2 diabetic erectile dysfunction: A protocol for systematic review and meta-analysis

**DOI:** 10.1097/MD.0000000000020665

**Published:** 2020-06-12

**Authors:** Fuhao Li, Xianliang Qiu, Hangyu Yao, Degui Chang

**Affiliations:** Hospital of Chengdu University of Traditional Chinese Medicine, Chengdu, Sichuan Province, China.

**Keywords:** erectile dysfunction (ED), protocol, type 2 diabetes mellitus (T2DM), vitamin D

## Abstract

**Introduction:**

Diabetic erectile dysfunction (DED) has gradually become a worldwide problem. Due to the mechanism of DED is not clear, it is impossible to treat it pertinently. Recently, some studies have shown that vitamin D is associated with DED, type 2 diabetes mellitus (T2DM) and erectile dysfunction (ED), but there is no systematic review and meta-analysis on the relationship between vitamin D and DED.

**Methods and analysis:**

The databases of English databases (PubMed, MEDLINE, EMBASE, Web of Science, Cochrane Library) and Chinese databases (China National Knowledge Infrastructure, China Biology Medicine Database, Wanfang Database, VIP Database) will be retrieved. The search strategy that will be run in the PubMed and tailored to the other database when necessary is presented in Table 1

**Table 1:**
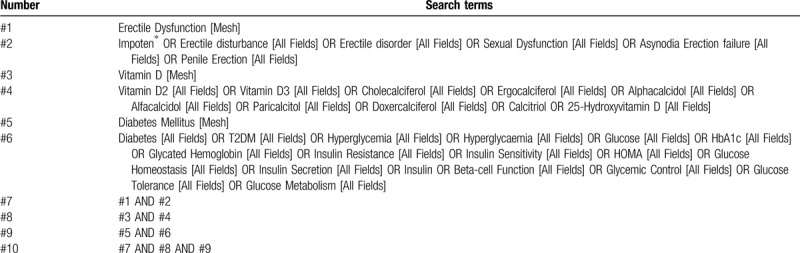
PubMed search strategy.

. RevMan 5.3 and Stata 11.0 will be used for Systematic Review and Meta-analysis. This protocol reported under the Preferred Reporting ltems for Systematic Reviews and Meta-Analyses Protocols (PRISMA-P) statement, and we will report the systematic review by following the PRISMA statement.

**Results:**

Through a systematic review, and meta-analysis when necessary, we can obtain the relationship between vitamin D and DED. We will share our findings in the third quarter of 2021.

**Conclusion:**

The association between serum vitamin D levels and type 2 diabetic erectile dysfunction will be assessed. Besides, the results of this review may provide some help for clinicians to make decisions.

**Ethics and dissemination:**

Ethical approval is not required as the review is a secondary study based on published literature. The results will be published in a public issue journal to provide evidence-based medical evidence for urologists and andrologists to make better clinical decisions.

**Protocol registration number:**

INPLASY202040164.

## 1 Introduction

Erectile dysfunction (ED) is a common male sexual dysfunction. It is defined as a persistent inability to acquire or sustain an adequate erection to engage in satisfactory sexual intercourse according to the International Consultation on Sexual Medicine.^[1]^ An epidemiological study showed the prevalence of ED is 19.2%,^[2]^ and other scholars considered that it will increase year by year, even some reviews predicted the global number of ED will reach 322 million by the year 2025.^[3]^ ED not only impairs male sexual confidence but also severely impacts the quality of life,^[4]^ meanwhile, it also imposes a heavy burden on family and public health service.

The pathophysiology of ED is multifactorial, however, it is mainly related to a reduction of endothelial function as a vascular disordered disease.^[5]^ Erections are primarily vascular events and the process owes to the release of neurotransmitters from the corpus cavernosa and the nitric oxide (NO), a relaxing factor, from the endothelial cells of the penis.^[6]^ Neurotransmitters and NO co-lead the corpus cavernosa to relax and allow blood to flow into the penis to expand it then sustain the erection. Hence, any disorder that may induce endothelial dysfunction (END) can interfere with vasodilatation, preventing the erections.^[7]^

Some research showed that the deficiency of 25-hydroxyvitamin D [25(OH)D] may cause END.^[8,9]^ Through genomic and nongenomic path-ways, 25(OH)D can protect endothelial cells against oxidative stress and inhibits apoptosis by increasing NO production in endothelial cells.^[10]^ So, 25(OH)D deficiency could be considered to raise ED.

Recently, several reviews also paid attention to the prevalence of diabetic erectile dysfunction (DED) due to diabetes has been considered to be one of the high-risk factors of ED and the majority approve that the incidence of DED is much higher than healthy men.^[11,12]^ It is reported that the male who have type 2 diabetes mellitus (T2DM) more frequently with a low level of testosterone and the majority presenting symptoms expressed as hypogonadism: about 25% of type 2 diabetic men may be occurred by hypogonadotropic (secondary) hypogonadism.^[13]^ The key mechanisms of type 2 diabetes-associated ED are considered as the decreased endothelial NO bioavailability and increased oxidative stress.^[14]^ Meanwhile, recent advances highlight that poor glycemic control and prolonged duration of diabetes in two-thirds of patients with diabetes and low vitamin D levels.^[15]^ Low serum 25(OH)D concentrations also have been found out to be associated with insulin resistance, obesity, glucose intolerance and fasting hyperglycemia.^[16–18]^ All these results can reflect the effects of 25(OH)D on glycemic control in diabetic patients.

Although the present studies consider that there is a certain relationship among ED, diabetes and 25(OH)D, we still need more large-sample and multicenter trials to further explore the mechanisms.

## 2 Objectives

This systematic review aims to integrate and assess the impact of vitamin D on ED and T2DM. Meanwhile, it will synthesize the correlation between the serum vitamin D level and type 2 diabetic erectile dysfunction (T2DED). Then, the role of vitamin D in the mechanism of T2DED will be performed in this paper and the results will provide better clinical decisions for the treatment of T2DED.

## 3 Methods

The protocol was registered on the International Platform of Registered Systematic Review and Meta-analysis Protocols (registration number: INPLASY202040164) which could be available on *https://inplasy.com*. The content refers to the statement of preferred reporting items for systematic review and meta-analysis protocols (PRISMA-P).^[19]^

### 3.1 Eligibility criteria

The inclusion and exclusion criteria are as follows.

#### 3.1.1 Types of studies

All the cross-sectional studies, cohort studies (including prospective and retrospective), and case-control studies will be included without publication status restriction or writing language. Case reports, narrative or systematic reviews, meta-analyses, letters, and other secondhand studies will also be excluded.

#### 3.1.2 Participants

Studies containing the comparison of serum Vitamin D levels among patients with DED, DM, ED and healthy people will be considered for inclusion. The race and age of participants will not be restricted in this review.

#### 3.1.3 Exposure

Patients with T2DED should meet the diagnosis of both T2DM and ED. T2DM should be diagnosed through comprehensive history collection, physical examination and related blood glucose tests under the diagnostic guidelines of the American Diabetes Association. Meanwhile, it is necessary to meet the clinical diagnosis of ED in patients with T2DM. The diagnosis can be diagnosed through comprehensive history collection, physical examination and even specific examination according to the diagnostic guidelines of the European Association of Urology, the American Urological Association, or other authoritative organizations.

The cutoff points of vitamin D deficiency and insufficiency are still a topic of discussion, we will follow Endocrine Society clinical guidelines and previously recommended cutoff points in diagnosing vitamin D deficiency when serum 25(OH)D was <20 ng/ml and vitamin D insufficiency when 25(OH)D was between 20 and 29.9 ng/ml.25 (OH) D is the main circulating vitamin D metabolite and a reliable indicator of vitamin D levels. The overall vitamin D level can be determined by detecting 25 (OH) D.

Patients with type 1 diabetes, prostate cancer, a history of prostatectomy and other diseases that can cause erectile dysfunction who have received or are currently receiving vitamin D replacement therapy were excluded from the study.

#### 3.1.4 Types of outcome measures

The main results of this study are investigating the concentration of vitamin D levels in subjects with and without DED and target at the 5-item version of the international index of erectile function (IIEF-5) score in subjects with (<20 ng/ml) and without vitamin D deficiency (>20 ng/ml).

The additional outcomes of this evaluation mainly focus on the association between DED and other biochemical tests, including plasma glucose levels, Hemoglobin A1c (HbA1c), free testosterone (FT), blood lipid profile (Triglycerides, total serum cholesterol, H-DL, L-DL levels), and so on. These clinical data will be recorded and used to explain the variability between studies if necessary.

### 3.2 Search strategy

#### 3.2.1 Data sources

Electronic databases will include English databases (PubMed, MEDLINE, EMBASE, Web of Science, Cochrane Library) and Chinese databases (China National Knowledge Infrastructure, China Biology Medicine Database, Wanfang Database, VIP Database). All the above databases will be searched from their inception to recognize related studies. The search strategy that will be run in the PubMed and tailored to the other database when necessary is presented in Table 1. Besides, the reference lists of review articles will be searched for any possible titles matching the inclusion criteria.

#### 3.2.2 Other sources of search

The researchers will also scan the database of Chengdu University of Traditional Chinese Medicine Library and our hospitals experts in endocrinology and urology will be consulted. Dissertations of degrees will be included. The WHO International Clinical Trials Registry Platform and Google Scholar will be searched for potential results. Besides, the ClinicalTrials.govregistry will be searched for any unpublished trials.

### 3.3 Data extraction, quality, and validation

#### 3.3.1 Study inclusion

**Figure 1:**
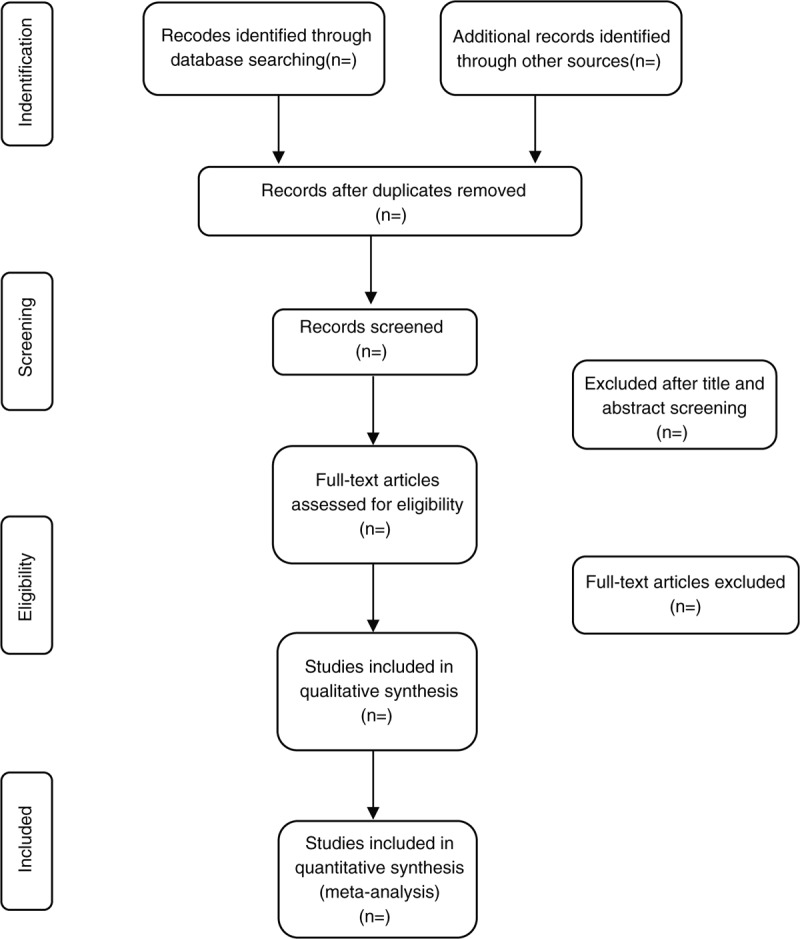
Study selection flow chart.

According to the eligibility criteria, the screening will be carried out in duplicate by 2 independent reviewers (XLQ & FHL) at all times. Studies will be removed if they do not meet the inclusion criteria. If the studies appear to meet the inclusion criteria or there is any uncertainty based on the information provided in the title and abstract, full texts will be obtained for further assessment. When necessary, we will contact the author for more details of the study to solve questions about eligibility. Disagreements will be resolved by discussion or taking the expert (DGC) for arbitration. The number and reasons for excluding trials will be recorded in detail. A flow diagram of the study selection is shown in Figure 1.

#### 3.3.2 Data extraction and management

The 2 independent reviewers (XLQ & FHL) will extract data from the included study. If there is any inconsistency between the 2 reviewers, the third member (HYY) will decide the outcome.

We will use a standardized data extraction table to extract the following information from the selected literature:

Publication information: title, first author, time of publication, country or region, and funding support.

Details of the methodology: participants, sample size, diagnostic criteria, demographic characteristics (including age, habit, ethnicity and education), recorded clinical research data (including plasma glucose levels, HbA1c, FT, blood lipid profile), data detection strategies, data analysis strategies, disease-related scales and their scoring results, data sources.

We will contact the author to obtain any missing information or solve related questions about the above data. If no clarification is provided after 4 weeks, the study will be included in the final analysis and the missing information will be marked.

### 3.4 Risk of bias assessment

Two reviewers (XLQ & FHL) will independently use different scales to assess the risk of bias based on the type of studies: cross-sectional studies will be assessed by the Agency for Healthcare Research and Quality (AHRQ) recommended criteria. The criteria include eleven items, answered by “yes”, “no”, “unclear”; case-control and cohort studies will use the Newcastle-Ottawa Scale (NOS) which assess the quality of studies with 8 questions in 3 broad categories:

1. patient selection;
2. comparability of study groups;
3. assessment of the outcome.

The evaluation will use the semi-quantitative principle of the star system and the highest score is 9 stars. Stars of 0–4 mean low-quality and 5–9 mean high-quality. Any disagreements will be solved by discussion or with arbitrament by the third reviewers HYY.

### 3.5 Quantitative data synthesis and statistical methods

#### 3.5.1 Data analysis and synthesis

Mata-analysis will be performed by the software RevMan 5.3. Our primary outcome will mainly investigate the concentration of vitamin D levels in subjects with and without DED. The secondary outcome will target at the IIEF-5 score in subjects with (<20 ng/ml) and without vitamin D deficiency (>20 ng/ml). We will take the standardized mean difference (SMD) to measure the difference of vitamin D levels or IIEF-5 scores and their corresponding 95% confidence intervals (95% CIs). We will assess heterogeneity with the χ^2^ goodness of fit and *I*^*2*^ statistics. Concerning *I*^*2*^, we will reference Cochrane recommendations. If the value of *P* < .1 or *I*^*2*^ < 40% that heterogeneity will be considered significant under the condition and the random-effects model is about to be employed. Otherwise, the fixed model will be conducted. In the case of the presence of heterogeneity, we will perform sensitivity analyses and retrogression when possible. Publication bias will be determined by a funnel plot and we will try to interpret funnel plot asymmetry by Egger linear regression test if funnel plots are asymmetric.

#### 3.5.2 Subgroup analysis and investigation of heterogeneity

If there is a sufficient number of studies, we will investigate potential sources of heterogeneity by performing subgroup analyses:

1. According to the type of testing: radioimmunoassay or immunoassay or some else.
2. The sample size of the included studies.
3. Some biomarkers which connected with this disease: plasma glucose levels, HbA1c, free testosterone, blood lipid profile (Triglycerides, total serum cholesterol, H-DL, L-DL levels), and so on.
4. The sociodemographic characteristics of participants: age, BMI, history of DM, current smoking, and alcohol status.

#### 3.5.3 Sensitivity analysis

If possible, a sensitivity analysis will be performed to test the reliability and stability of the review result and to detect the source of heterogeneity. This can be done by excluding trials with a high risk of bias or eliminating each study individually. Then the analysis will be repeated after the exclusion of low methodological quality studies.

#### 3.5.4 Grading the quality of evidence

Grading of Recommendations Assessment, Development and Evaluation (GRADE) method will be performed to evaluate the level of confidence in regards to outcomes. Two independent reviewers (XLQ & FHL) will assess these studies. In most cases, disagreements were resolved by discussion between the 2 reviewers. If disagreement remained after discussion, the third reviewer (HYY) will be consulted before taking the final decision on the disagreements.

#### 3.5.5 Publication bias

Published bias will be measured by the funnel plot. If the result is indistinct, the Begg test and Egger test will be used (by STATA software 11.0).

## 4 Discussion

ED is a multifactorial disease, and its mechanism of etiology and pathogenesis is not yet clear. As a common male disease, ED not only seriously affects the physical and mental health of men, but also greatly harms the feelings between patients and partners, and exerts great burden on public health. DED is one of the common complications of diabetes, the pathogenesis is more complex, and the degree of ED in DED patients is higher, so it is more difficult to treat. In recent years, many scholars believe that there may be a certain correlation between Vitamin D and DED, but it has not been scientifically and systematically evaluated. The purpose of this study is to assess whether there is a correlation between Vitamin D and DED and to try to explore the possible pathogenesis, and we hope that this study will provide higher-quality evidence. There are some limitations to the review. Different duration and severity of the disease may lead to the risk of heterogeneity. Besides, the number of articles studying the correlation between Vitamin D and DED is small, and the sample size is low at present, which may lead to a lack of reliability.

## Author contributions

**Conceptualization:** Fuhao Li.

**Data curation:** Xianliang Qiu, Hangyu Yao.

**Formal analysis:** Xianliang Qiu, Fuhao Li.

**Methodology:** Fuhao Li.

**Project administration:** Fuhao Li, Degui Chang.

**Software:** Xianliang Qiu, Hangyu Yao.

**Supervision:** Degui Chang.

**Validation:** Xianliang Qiu.

**Writing – original draft:** Fuhao Li, Hangyu Yao.

**Writing – review & editing:** Fuhao Li, Degui Chang.

DC is the guarantor. All review authors critically reviewed, revised, and approved the subsequent and final version of the protocol.
